# Determining the Right Levels of Health Coaching and Heart Rate Variability Biofeedback in a Workplace Behavior Change Intervention: Multiphase Optimization Strategy Preparation Study

**DOI:** 10.2196/47181

**Published:** 2024-02-14

**Authors:** Sean Locke, Jenna Osborne

**Affiliations:** 1 Department of Kinesiology Faculty of Applied Health Sciences Brock University St Catharines, ON Canada

**Keywords:** mobile health, mHealth, behavior change, stress management, intervention, pilot study, heart rate variability, health coaching, coach, coaching, coaches, work-related stress, stress, wellness, burnout, behavioral intervention, work, worker, workers, employee, employees, occupational health, job, satisfaction, web-based, remote, corporate, web analytics, biofeedback, survey, surveys, interview, interviews, experience, experiences, attitude, attitudes, opinion, opinion, perception, perceptions, perspective, perspectives, acceptance

## Abstract

**Background:**

Work-related stress is associated with poor job performance and negative health outcomes. Changing health behaviors through corporate wellness programs can improve physical and mental health and help employees manage stress. This project sought to pilot the potential addition of brief coaching and biofeedback to an 8-week web-based self-help program to improve employee stress using the multiphase optimization strategy.

**Objective:**

This study aims to determine which candidate components will be tested in a later optimization phase and at what dose they will be tested, examine the feasibility and acceptability of delivering the different components, investigate whether the outcomes can be feasibly measured, and review evidence to build a conceptual model before the optimization phase.

**Methods:**

The study was positioned within the preparation phase of the multiphase optimization strategy. It is a 2×2×2×2 design with 4 components: 2 types of health coaching and 2 types of biofeedback. All components were tested by turning them *on* or *off*. A total of 16 adult office workers (mean age 40, SD 14.3 years; n=15 women) completed an 8-week self-paced web-based stress management and health behavior change program and were randomly assigned to 1 of the 16 conditions, created from a combination of the 4 candidate components. Assessments included web analytics, surveys, and interviews regarding program recommendations, likes, and dislikes.

**Results:**

Findings from the interviews provided suggestions to improve the intervention (eg, separating wellness from stress content) and trial conduct (eg, streamlining the onboarding process). On average, participants logged into the wellness program 83 times (range 36-291), with 75% (12/16) participant retention and 67% (8/12) survey completion. There were no reported problems with coaching or obtaining data from interviews or apps. The interview findings suggested potential mediators to include and assess in a future conceptual model.

**Conclusions:**

The results provided areas to improve the intervention content and trial methods. Instead of progressing to the next scheduled large-scale optimization phase, our plan to iterate through a second preparation phase after making changes to the protocol, apps, and corporate coaching partner.

## Introduction

### Background

Work is one of the major contributors to chronic stress. Work-related stress is experienced by most employees and is related to poor job performance and lower job satisfaction [1,2]. Chronic stress, a common outcome of workplace stress, is a critical risk factor for a variety of negative health outcomes [1,3] and is linked to the development of cardiovascular disease and depression [4-6]. Meta-analytic findings from a study conducted among >80,000 employees demonstrated an increase in coronary heart disease risk for those experiencing work stress (risk ratio 1.16) [7]. There are considerable costs to the employer from unmanaged high stress, resulting in performance decreases and absenteeism [8]. These costs can be ameliorated through employer-funded health and wellness programs [9,10], which can also serve as feasible strategies for chronic disease prevention [11].

There are numerous psychological, social, and physiological benefits of engaging in positive behaviors such as physical activity and healthy eating [12-14]. It is well established that regular engagement in positive health behaviors (eg, exercise and diet) is associated with lower stress [15-17]. Despite the potential benefits, only a few individuals meet recommendations, missing out on these benefits [18,19]. Individuals spend as much as half of their waking hours working, and the workplace offers an opportunity to promote health, including stress management. Behavior change interventions target exercise and diet in the workplace can improve health behaviors [20-24]. Evidence from a review of 23 studies indicates that workplace interventions can improve physical and mental health outcomes [25].

Internet-based interventions represent a scalable approach to health promotion. High rates of internet use and the convenience of accessing content 24 hours a day allow intervention to reach participants, whether they are at work or home [26]. Web-based self-help interventions are structured such that the participants can complete these at their own pace and do not require direct contact with an interventionist. A review of 23 web-based self-help interventions reported significant weight loss across studies [27]. Workplace health and wellness programs are often multifaceted [28]; web-based self-help approaches may be particularly useful for delivering program components such as health information or behavior change strategies designed to help individuals learn about new behaviors and plan their behavior change efforts.

Behavior change techniques (BCTs) represent the active ingredients of an intervention [29]. BCTs are used to target the hypothesized mechanism of action to help individuals change their behaviors. For example, goal setting and self-monitoring are among the most common BCTs in physical activity and diet interventions [30]. These BCTs target and seek to improve individuals’ confidence and ability to self-regulate their behavior. These types of BCTs are suitable for internet-based delivery, given the ease of use and accessibility of websites and apps.

Individual health and wellness coaching can be an effective way to promote behavior change for a variety of health behaviors [31-33]. Motivational interviewing is a person-centered coaching approach to enhance clients’ intrinsic motivation for change [34]. Motivational interviewing is a heart- and mindset approach to coaching that aims to embody compassion, acceptance, partnership, and evocation with the client [34]. There are 4 processes and 4 communication skills to support the spirit of motivational interviewing. The 4 processes (ie, engaging, focusing, evoking, and planning) are designed to guide clients from a state of ambivalence toward a state of willingness to make a healthy change. The 4 communication skills (ie, open-ended questions, affirmations, reflections, and summaries) are fundamental to reflective listening and are ways to demonstrate that spirit. Motivational interviewing–based interventions have consistently been shown to be effective across numerous health behaviors (eg, diet, exercise, and oral health) [35]. Even brief motivational interviewing–based interventions can be effective in improving diet and exercise [36]. Brief motivational interviewing coaching may be a scalable approach for providing health coaching in workplace interventions. Furthermore, individual-level coaching represents a common strategy that multifaceted health promotion programs use in the workplace [28]. A recent meta-analysis revealed that physical activity interventions highlighted biofeedback as 1 of the 4 BCTs related to the effective initiation of physical activity [37]. Heart rate variability (HRV) is a reliable means of acutely and indirectly measuring stress through the autonomic nervous system [38,39]. It has also been used as an index of physical and emotional health [40-42]. HRV biofeedback (HRV-BF) is a noninvasive method for delivering HRV data in real time to the desired user [43,44]. HRV-BF has been shown to be effective across different settings for improving HRV and other psychological and physiological outcomes [45,46]. HRV-BF has been shown to be effective in the workplace [47,48] and may help promote self-awareness and allow individuals to better regulate their psychological and physiological states [49]. HRV-BF may be a beneficial tool alongside behavioral intervention components to promote awareness and self-regulation.

We partnered with CoreHealth, a company that supplies corporate health and wellness coaches with an web platform to deliver a variety of wellness tools and interventions, including coaching, to their clients. This project sought to pilot the potential addition of brief coaching and HRV-BF to an 8-week web-based self-help program using the multiphase optimization strategy (MOST [50]).

### MOST: Preparation Phase

MOST will be used to pilot this study, to optimize, and evaluate the efficacy of the stress management and health behavior change intervention to improve workers’ stress and well-being. MOST is a 3-phase framework that aims to develop interventions that are effective, efficient, economical, and scalable [50]. The 3 phases include *preparation* (ie, selecting which intervention and at what level components are feasible to examine), *optimization* (ie, determining the optimized package of components via an optimization randomized controlled trial), and *evaluation* (ie, evaluating the efficacy of the optimized package through an evaluation randomized controlled trial). The factorial design of MOST in the optimization phase will allow us to examine the effect of adding intervention components to a standard 8-week behavior change program. This paper reports the first phase of MOST, the preparation phase, using the reporting recommendations by Landoll et al [51], with the following four study objectives:

1. Determine which candidate components will be tested in a later optimization phase and at what dose they will be tested
2. Examine the feasibility and acceptability of delivering the different components
3. Investigate whether the outcomes can be feasibly measured
4. Review evidence to build a conceptual model before the optimization phase.

## Methods

### Design Overview

This study is registered with ClinicalTrials.gov (NCT05150574). In this phase, the components are first tested with a few participants to identify implementation problems and refine them to ensure acceptability and feasibility. The proposed future optimization phase will use a 2^4^ (2×2×2×2) factorial experiment to test all combinations of the 4 candidate components by turning them “on” or “off,” resulting in 16 different conditions in total (Table 1).

**Table 1:** Combination of intervention components comprising the 16 intervention conditions.

Number	Components^a^				
	8-week program	Daily resting HRV^b^ feedback	Momentary HRV feedback	Behavioral initiation coaching	Practice with feedback coaching
1	On	Off	Off	Off	Off
2	On	Off	Off	Off	On
3	On	Off	Off	On	Off
4	On	Off	Off	On	On
5	On	Off	On	Off	Off
6	On	Off	On	Off	On
7	On	Off	On	On	Off
8	On	Off	On	On	On
9	On	On	Off	Off	Off
10	On	On	Off	Off	On
11	On	On	Off	On	Off
12	On	On	Off	On	On
13	On	On	On	Off	Off
14	On	On	On	Off	On
15	On	On	On	On	Off
16	On	On	On	On	On

^a^All participants will have access to the content on CoreHealth, and they will be randomized to receive 1 of the 16 conditions.

^b^HRV: heart rate variability.

For this study, we recruited 16 participants, 1 per condition. There are four candidate coaching components being delivered, with a fifth untested component that all participants will receive the following:

1. *Daily resting HRV biofeedback*: the participants will be asked to record regular HRV measurements in the morning before eating or drinking.
2. *Momentary HRV biofeedback*: throughout the workday, the participants will be asked to take in-the-moment HRV measurements, with the option of performing a mindfulness activity to manage elevated stress.
3. *Health coaching and behavior change initiation*: the participants will receive one 15-minute wellness coaching session at the start of week 1 of their program.
4. *Health coaching and practice with feedback coaching*: the participants will receive one 15-minute wellness coaching follow-up session in either week 2 or week 4.
5. *Web-based behavior change program (all participants receive)*: this is an 8-week web-based program that provides the participants with access to 8 weekly behavior change and stress management modules. The platform also contains information on a variety of health behaviors.

### Ethical Considerations

This study received ethics approval from the Brock University Research Ethics Board (REB #20-367) before recruitment. Prospective participants were emailed information about the study, and those interested were directed to a link to complete the eligibility questionnaire via Qualtrics (Qualtrics International Inc). Eligible participants provided informed consent on Qualtrics before scheduling an onboarding meeting with study staff. The health coach also provided informed consent to be interviewed. All data were electronic, deidentified, and stored on the institutional servers of the corresponding authors. There was no compensation for participating in this study. Generative artificial intelligence was not used in any part of the manuscript writing.

### Participants and Procedure

A total of 16 participants were recruited for the study. The eligibility criteria included being an adult aged ≥18 years, being able to read and comprehend English, having an Apple iPhone 8 or newer version (iOS 14 or newer) or an Android (operating 7.0 or newer), having a self-reported BMI <40 kg/m^2^, and reporting at least a moderate amount of daily job stress. We recruited potential participants through email blasts and word of mouth from companies primarily comprising office workers. Interested participants were directed to a web link to determine their eligibility and to provide informed consent. The participants were contacted to schedule a 30-minute onboarding phone call to discuss study procedures and help them set up and learn about the study apps. Following the onboarding call, the participants were asked to complete the baseline survey measures and record baseline HRV for 7 days before starting the 8-week study. For the first 3 days, HRV was measured when the participants woke up in the morning, to be averaged as before and after the program to assess change, whereas for the following 4 days, they were to get accustomed to taking different HRV readings. If requested, the participants were sent reminders to record their baseline HRV readings.

At the end of 8 weeks, the participants were interviewed to discuss likes and dislikes and suggested improvements to the study. At the end of the interview, they were asked to complete a poststudy survey and record 3 more poststudy HRV readings. Furthermore, the wellness coach was interviewed at the end of the study. All interviews were conducted via Microsoft Teams (Microsoft Corp). Interviews were recorded, and written transcripts of the interviews were created. In response to ongoing participant feedback, we made a deviation to the study protocol, which received research ethics approval, and conducted a 15-minute interview approximately halfway through the 8-week program. This was sparked by a few participant emails regarding the onboarding process and the early technical issues. We chose to interview the participants about these challenges while the issues were fresh in their mind rather than waiting for another 4 weeks to ask questions at the poststudy interview.

### Candidate Intervention Components

There are 5 components being delivered. Four of the components will be tested and examined as part of a future factorial experiment: (1) daily resting HRV biofeedback, (2) momentary HRV biofeedback, (3) behavioral initiation coaching, and (4) practice with feedback coaching. The fifth is a constant component with all the participants receiving an 8-week behavior change and stress management program.

#### HRV Biofeedback

Light Heart is a mobile phone app (CoreHealth) that was recently designed and developed by CoreHealth to be used as a supplementary tool in their web-based platform to provide HRV biofeedback (BCT 2.6 biofeedback). Research assessing the concurrent validity of the app in assessing pulse intervals against a gold-standard electrocardiogram (BioAmp FE132, ADInstruments) is currently being reviewed. The results demonstrated a strong positive linear correlation (*r*=0.99; *P*<.001) between the Light Heart app and the electrocardiogram. The app was designed to measure and provide HRV biofeedback through photoplethysmography to detect changes in blood flow through the skin. All the participants were asked to download the app to measure their HRV. The participants were then asked to connect to this app via a *mobile access code* created through the CoreHealth platform. The participants could obtain an HRV reading by opening the app, selecting “take reading,” placing their finger over their rear phone camera, and flashing for 60 seconds. The participants were given a visual prompt if they were moving their fingers too much to obtain a clear reading.

Because HRV metrics are complex to understand (eg, SD of the normal sinus beats [sdNN; ms]), the participants view a user friendly HRV index. The proprietary index uses a 0 to 100 scale to graphically represent an individual’s HRV, with higher scores indicating poorer HRV. To assess their baseline HRV, the participants were instructed to find a quiet, distraction-free place to sit and measure their HRV when they first wake up in the morning and before they eat or drink. There are two forms of HRV biofeedback being tested: (1) momentary and (2) daily resting.

Those receiving *momentary HRV biofeedback* were instructed to use the app to record in-the-moment HRV measurements throughout the day when they experienced elevated stress. Higher HRV scores indicated elevated stress. In these instances, the participants were recommended to take a few minutes to perform a mindfulness or meditation activity, following 1 of the videos provided on the CoreHealth platform. Following the activity, the participants were prompted to take another HRV measurement to determine whether their HRV improved after performing the stress management activities.

Those receiving *daily resting HRV biofeedback* were asked to regularly obtain HRV measurements each morning, similar to recording their baseline HRV. In this way, a regular morning measurement may be an indicator of improved HRV over time as a result of making healthy changes known to impact HRV (eg, improved cardiorespiratory fitness, sleep quality, and mindfulness).

#### Health Coaching: Behavioral Initiation and Practice With Feedback (BCT 3.1, Social Support-Unspecified)

All coaching for this study was conducted through CoreHealth’s videoconferencing tool or via telephone. The wellness coach was trained in a communication style called motivational interviewing. Motivational interviewing is a person-centered collaborative counseling style that helps clients strengthen their autonomous motivation and commitment to change [34].

Coaching at 2 different points in the intervention was tested: behavioral initiation coaching and practice with feedback coaching. In this study, those who received coaching components received motivational interviewing–based health and wellness coaching. Consistent with the spirit of motivational interviewing and standard practice for many of CoreHealth’s clients, the participants were given the autonomy to choose the behavior they wished to change. Those receiving *behavioral initiation coaching* received one 15-minute behavioral coaching session to start week 1 of their program. In this session, the participants established their commitment to change, decided on a health behavior change goal, and were asked to create an action plan and schedule and track their progress using CoreHealth. Those receiving *practice with feedback coaching* received a follow-up 15-minute session. In this session, clients discussed their level of success in achieving their goals, revised their goals as necessary, and discussed potential barriers to achieving their goals. For this preparation phase, we investigated the 2 different time points to deliver the follow-up coaching at either 2 or 4 weeks after the initiation of counseling to determine which was more acceptable to the coaches to facilitate behavior change.

#### Web-Based Behavior Change Program

The CoreHealth wellness platform was used to deliver the 8-week web-based behavior change program. In consultation with CoreHealth, BCTs used in the current 8-week period consisted of prompts (eg, email reminders to check-in; BCT 7.1 [29]); goal setting, action planning, and self-monitoring tasks (eg, prompting to create a goal and then providing a calendar to schedule and track participants’ behavior; BCT 1.1, 1.3, 1.4, and 2.3); instruction on how to perform a behavior (eg, videos on how to meditate and examples of different types of exercise; BCT 4.1 and 6.1); reframing (eg, suggesting new perspectives to view barriers; BCT 13.2); providing health-related information (eg, text, video, infographics, or others such as recipes and guidelines; BCT 5.1, 5.3, and 5.6); and confidence ruler (motivational interviewing technique 11 [52]). Refer to Table 2 for a breakdown of the intervention content by week. Furthermore, the participants had access to modular health information and videos on the platform. Health information covered a variety of health and wellness behaviors. A total of 5 wellness areas were included on the study platform in consultation with CoreHealth, chosen based on their association to HRV (physical activity, sleep, meditation and mindfulness, diet, and stress management [53]). The intervention content was based on existing CoreHealth behavioral and stress management programming. The participants were asked to download the CoreHealth app, which provided the same features as the web platform but in an app form.

**Table 2:** Outline of the weekly content of the 8-week CoreHealth program that all the study participants received.

Week	Behavior change content	Stress management content
1	Set SMART^a^ long-term goal Prompt reflection of values underlying the behavior change Set a 1-week action plan for health and wellness behavior Confidence ruler	None
2	Review previous action plan and adjust or set a new 1-week plan Overcoming barriers Confidence ruler	None
3	Review previous action plan and adjust or set a new 1-week plan Set a stress management goal Confidence ruler	Providing information for defining stress Introduction to platform stress resources Introduction to mindfulness meditation
4	Review previous action plan and adjust or set a new 1-week plan Confidence ruler	Reframing your stress response Being kind to yourself in stressful situations
5	Review previous action plan and adjust or set a new 1-week plan Confidence ruler	Cognitive defenses Disputing negative self-talk Keeping stress in perspective Shifting focus forward Avoiding blame game Being grateful
6	Review previous action plan and adjust or set a new 1-week plan Confidence ruler	Ways to prevent stress Identifying and eliminating sources of stress Replacing negative coping mechanisms with positive ones Placing stress-relieving habits into easily accessible forms Boosting stress immunity with regular physical and mental exercise Making a little plan (1-week plan) Making a big plan (long-term goal)
7	Review previous action plan and adjust or set a new 1-week plan Confidence ruler	Steps to avoid unnecessary stress Planning the day Organizing the surrounding and tasks Building time management skill Problem-solving and strategizing to manage daily life stress
8	Reflect on the past content and set a plan to maintain positive health habits Review the previous action plan and adjust or set a new 1-week plan Confidence ruler	Reflect on the past content and set a plan to maintain positive stress management habits

^a^SMART: specific, measurable, attainable, realistic, and timeframe.

### Measures

Refer to Tables 3 and 4 for a list of the study measures and participant interview outline.

**Table 3:** List of the study measures and time of assessment.

Measure name	Study measures				Time points (before and after program or after program only)
	Concept being measured	Number of items	Example item		
Hospital Anxiety and Depression Scale [54]	Self-measured instances of anxiety and depression	14 items and scale range of 1 (not at all) to 4 (yes, all the time)	“I feel tense or wound.”	Before and after	
Job Stress Scale [55]	Describes possible feelings and emotions felt during time spent at work or because of time spent at work	5 items and scale range of 1 (strongly disagree) to 5 (strongly agree)	“A lot of time my job makes me very frustrated or angry.”	Before and after	
Psychological Well-Being Scale [56]	Assesses participant’s current level of well-being	9 items and scale range of 1 (strongly disagree) to 7 (strongly agree)	“I judge myself by what I think is important, not by the values of what others think is important.”	Before and after	
Self-rated health scale [57,58]	Self-reported health scale	1 item and scale range of 1 (excellent) to 5 (poor)	“In general, over the past four weeks would you say your overall health is”	Before and after	
Self-regulatory efficacy for health behavior goals [59,60]	Rating confidence levels to make health behavior change	8 items and scale range of 0% (not confident at all) to 100% (completely confident)	“How confident are you that you will develop solutions to cope with unexpected barriers that can interfere with achieving your health goals?”	Before and after	
Wellness Behaviors Inventory [61]	Self-reported questions on how regularly participants engage in different health and wellness behaviors	17 items and scale range of 1 (<1 time a week or none) to 5 (every day of the week)	“I walk as much as possible. ie, taking stairs instead of elevator etc.”	Before and after	
Motivation Scale	The extent to which the participant believes the program received motivated change to their stressor health behaviors	5 items and scale range of 1 (not at all motivating) to 5 (extremely motivating)	“To what extent did receiving biofeedback help motivate your healthy changes? (if applicable)”	After only	
Acceptability measures [62]	Acceptability and feasibility of intervention measures	8 items, including 4 for acceptability and 4 for feasibility, and scale range of 1 (completely disagree) to 5 (completely agree)	“This program seems easy to use.”	After only	

**Table 4:** Semistructured interview outline.

Interviewee	Interview questions	Time points (before and after program or after program only)
Participants	Onboarding process How did you find the process of getting enrolled and starting the study? What was helpful about getting started in the study? What was challenging about getting started? What do you think would improve the process of starting the study? How did you find the process of downloading the apps and logging in? What do you think would improve the process of downloading and logging into the apps? CoreHealth platform and content What did you like about the platform? What did you not like about the platform? Which modules did you use on the CoreHealth platform? What did you think of the information that you were given? To what extent did the content help you make changes to your health or stress management? Was there anything you would change about the platform? How can we improve it? Light Heart Content (if applicable) What did you like about Light Heart? What did you dislike about Light Heart? Did you encounter any barriers to measuring your HRV^a^? Please describe them. Was there anything you would change about the app? How can we improve it? Health coaching (if applicable) Tell me about your experience working with your coach? What did you like and dislike about it? Did the coaching help you make healthy changes? If so, how? What could be improved?	After only
Health coach	Tell us about your experience delivering health coaching to our study participants? What did you like about the format of the study’s health coaching? What did you dislike about the format of the study’s health coaching? What did you think about the different conditions of the research study? Tell us what do you think needs to change about the study format to help participants elicit more health positive change? Do you have any general feedback about coaching and study format?	After only

^a^HRV: heart rate variability.

### Analysis

Descriptive statistics were computed using SPSS software (IBM Corp). The 6-phase method proposed by Braun and Clarke [63] was used to inductively analyze the interview data. Interview data were transcribed verbatim. A total of 2 raters read through the responses to become familiar with the data; main codes and subcodes were identified, using reflexive thematic analysis and then exemplar quotations for each code were selected.

## Results

### Sample Characteristics

A total of 16 adults consented to participate and were randomized to each of the 16 study conditions. The sample consisted of 15 women and 1 man (mean age 40.9, SD 14.3 years). All the participants worked full-time and had a mean BMI of 24.03 (SD 4.75) and mean job stress of 2.66 (SD 0.5). Regarding race, of the 16 participants, 15 (94%) identified as being White and 1 (6%) as being Southeast Asian. Of the 16 participants, 15 (94%) were married or living as married and 1 (6%) did not respond. Of the 16 participants, 11 (69%) reported having a bachelor degree or college diploma, 3 (19%) reported having postgraduate degrees, and 2 (12%) did not respond.

### Participant Retention

A total of 4 (25%) of the 16 participants dropped out of the study before completing the 8-week program. Reasons for study dropout included pregnancy (1/16, 6%), life stress unrelated to the study (2/16, 12%), and no stated reason (1/16, 6%). Of the 12 participants who completed the program, 8 (75%) completed the poststudy follow-up assessment. Regarding item-level missing data from complete surveys, there was 8.3% missing survey data in the prestudy measures and 15.6% missing survey data in the poststudy measures.

### Participant Engagement and Treatment Fidelity

A total of 12 (75%) out of the 16 participants completed the entire 8-week program, 1 (6%) completed 7 weeks, and 3 (19%) completed ≤3 weeks. Participants logged into the web-based CoreHealth platform an average of 83 times during the study (SD 63.8; range 36-291 log-ins). The CoreHealth program log-ins by group were as follows: daily resting HRV (mean 110.1), momentary HRV feedback (mean 70.7), initiation coaching (mean 64.4), and feedback coaching (mean 90.6). Among the 12 participants initially randomized to a condition that required the use of Light Heart throughout the program, the average number of HRV readings during the 8-week program was 12.55 (range 0-39), suggesting that the participants recorded an HRV reading with an average of 1.80 readings per week. Of the 4 (25%) participants not randomized to use the Light Heart app during their 8-week program duration, 3 (75%) did not use Light Heart and 1 (25%) used it once during the program. All the participants received the coaching they were randomized to. The average acceptability score was 3.9 (SD 0.66), and the average feasibility score was 3.78 (SD 0.91).

### Trial Conduct Interview Findings

Although more participants found the trial onboarding process helpful, there were few suggestions for improving the trial methods (Table 5). The most frequently reported code within the trial conduct was the usefulness of the onboarding process (9/16, 56%). However, 4 of the participants reported that the onboarding process could be improved. For example, providing videos in addition to showing how to use the apps in the onboarding call would help participants proceed with the trial when they went on their own. In total, 2 of the participants found that the program length of 8 weeks was sufficient, whereas 4 of the participants mentioned that a shorter program would better fit their behavior change efforts. Informal feedback from CoreHealth staff suggested that it might be difficult to manage the backend for 16 conditions and suggested fewer conditions moving forward.

**Table 5:** Participant interview findings related to trial conduct.

Code		Definition	Illustrative quote
**Likes, 2 codes**			
	Onboarding process, 9	Perceived like or dislike of the study’s enrollment process	“Using the Light Heart app was challenging but it was helpful to have the onboarding call and having someone to reach out to*.”* [HRT101]^a^
	Program length, 2	Perceived like or dislike of the 8-week program duration	“But I think 8 weeks, that’s a good time to actually make changes and then assess those changes*.”* [HRT101]
**Dislikes, 2 codes**			
	Onboarding process, 4	Perceived like or dislike of the study’s enrollment process	“I found it a little bit difficult is that some of the instruction were in a PDF and others were in that email. So you know flip flipping from different pieces of information. And I was like if it was like you know all into the same document so that I know one was for my code*.”* [HRT108]
	Program length, 4	Perceived like or dislike of the 8-week program duration	“Personally, I think a four to six weeks. I understand 8 weeks kind of helps you get in the habit of doing it, but after six weeks I’ve almost kind of forgot about it*.”* [HRT111]

^a^HRT denotes participant number.

### CoreHealth Interview Findings

There were 8 positive and 4 negative codes associated with the use of the CoreHealth platform (Table 6). A total of 6 different BCTs were coded 17 times as being positive for the program, which included prompts, self-monitoring and planning, instruction on how to perform a behavior, reframing, health-related information, and a confidence ruler. Participants noted that they enjoyed the platform features such as a weekly outlook of the self-paced modules. They also liked the health-related information on mindfulness and stress management, particularly the 10-minute videos (although 3 suggested having shorter video options available). However, the action planning feature of the weekly program drew critical feedback (n=10). Although some did not find any benefit in setting an action plan, others found that it required too much writing and that completing the action plan became repetitious as there was no function to copy the plan details from the previous week. The overall usability of the platform interface received both positive n=5 and negative reactions n=11. Most usability issues surrounded updating the user interface, including streamlining the action planning function. Additional instructions for navigating the platform were suggested 3 times for improving the user experience (eg, how-to-use videos).

**Table 6:** Participant interview findings related to the 8-week program and the Core Health platform.

Code		Definition	Illustrative quote
**Likes, 8 codes**			
	BCTs^a^: 17 Subcodes: Confidence ruler: 1 Prompts: 2 Self-monitoring: 7 Instructions: 1 Reframing: 1 Health-related information: 5	Confidence ruler: rating scale used to help quantify patient readiness to improve their condition Prompts: introducing or defining environmental or social stimulus with the purpose of prompting or cueing the behavior Self-monitoring and planning: establishing a method for the person to monitor and record their behaviors as part of a behavior change strategy Instruction on how to perform the behavior: advising or agreeing on how to perform the behavior Reframing: suggesting deliberate adoption of a perspective or new perspective on behavior (eg, its purpose) to change cognitions or emotions about performing the behavior Health-related information: useful content linked to health positive behaviors, such as stress management, mindfulness, and sleep quality	Confidence ruler: “I really liked the scale. Like how confident you feel about doing it? Love that. And it wasn't too, you know, one to 10. I think it was like one to five.. So it was really easy to... you don’t get too many choices you don’t know.” [HRT101] Prompt: “Having like push notifications or like something else that reminds you that you’re in this program.” [HRT113] Self-monitoring and planning: “I liked that I got kind of the weekly outlook and that you could see what the week was.” [HRT107] Instruction on how to perform the behavior: “I liked the breathing exercises...even guided meditations, I like much better than the one that you just. You know you kept yourself.” [HRT106] Reframing: “Like the like reframing, negative responses, and stuff. And like I definitely see the value.” [HRT113] Health-related information: “I liked the breathing exercises...even guided meditations, I like much better than the one that you just. You know you kept yourself.” [HRT106]
	Module length: 2	Perceived like or dislike of the amount of time to complete the action plan	“I thought it was good. I thought it was concise and you know I don’t like things that go on too long and that are too wordy.” [HRT111]
	Usability: 5	Perceptions of the capacity of the system to perform the tasks safely, effectively, or efficiently while enjoying the experience	“I liked the content, it was user friendly like on mobile devices.” [HRT101]
**Dislikes, 4 codes**			
	Length of stress management videos: 3	Perceived like or dislike of the video duration	“The only issue I had with them. I think there were a little too long so if you could just do like a you know instead of doing the full 10 minutes let me just do it for three or four minutes.” [HRT106]
	BCT Action Planning: 10	Action planning: participants found that weekly action planning required too much writing and commitment	“I found sometimes I end up saying a lot of the same things or like it was something that was out of my control and I couldn’t change....” [HRT107]
	Usability: 11	Perceptions of the capacity of the system to perform tasks safely, effectively, or efficiently while enjoying the experience	“The I guess it’s not the cleanest looking app, but it was functional...” [HRT106]
	Quality of instructions: 3	Perceived quality or clarity of the instructions	“Step by step document or personalized calendar would be nice so that participants can see what I have to do on each day would be helpful” [HRT129]

^a^BCT: behavior change technique.

### Light Heart Interview Findings

There were 8 possible codes regarding Light Heart from the interviews (2 likes and 6 dislikes; Table 7). There were both positive n=6 and negative n=8 feedback about the usability of the app. Participants across both the daily resting and momentary HRV components generally liked the look and feel of the app. Feedback about technical issues did not differ across participants receiving either HRV component as the issues were not component specific. Technical issues of poor signal quality, failed access codes early in the program, and elevated light temperature on some Android phones muted enthusiasm for the app in a few participants. Some participants noted that additional instructions about using and troubleshooting the app would enhance app usability. Suggestions included providing more detailed in-app explanations for failed readings owing to poor signal quality and providing more demo videos on how to use the app (eg, finger placement based on the phone and number of phone cameras).

The participants generally enjoyed recording their HRV readings to obtain biofeedback (11/16, 69%), which primed them to think about managing their stress or health and wellness goals. One participant remarked about obtaining their HRV as follows:

There was a day I felt pretty stressed out so I tested using the HRV and then I did a breathing exercise. I tested again and it had improved. It’s validating to see those numbers. It makes you feel better to measure again and see that it worked.

HRT104

One participant who was randomized to using both types of HRV-BF remarked, “the best usage for HRV is really [pre-post] biofeedback,” suggesting a preference for momentary HRV (HRT108). All participants had access to both types of readings: pre- and postfeedback readings used in the momentary HRV condition and daily resting readings. This confused participants, regardless of the condition, who were uncertain about which type of reading they should record and when.

**Table 7:** Participant interview findings related to obtaining heart rate variability (HRV) biofeedback.

Interview findings and code		Definition	Example quotes
**Likes, 2 codes**			
	Usability: 6	Perceptions of the capacity of the system to perform tasks safely, effectively, or efficiently while enjoying the experience	“It’s nice not having to keep up with different Instruments.” [HRT101]
	Biofeedback: 11	Biofeedback: provide beneficial feedback about the body (eg, physiological or biochemical state) using an external monitoring device as part of a behavior change strategy	“The best usage for HRV is really biofeedback; part of managing stress and finding ways to calm down.” [HRT108]
**Dislikes** **,** **6 codes**			
	Reading length: 2	Perceived like or dislike of the amount of time required to take an HRV reading	“If the readings didn’t take a whole minute” in response to how they would use Light Heart differently [HRT104]
	Quality of instructions: 5	Perceived quality or clarity of the instructions	“I don’t think there was enough instructions in the app to use it properly. Wasn’t sure how to stop the readings from quitting so maybe a demo video and a little trigger.” [HRT113]
	Technical problem: 21 Subcodes: Signal quality: 10 Access code: 8 Temperature: 3	Signal quality: capability of the HRV measure to obtain a successful reading Access codes: numeric code used to link the study’s mobile apps to the CoreHealth platform Phone temperature: perceived temperature of the flash while taking an HRV reading	Signal quality:“It was it was difficult to get a reading, and many mornings I would have to try multiple times” [HRT104] Access codes:“It was difficult to connect the apps” [HRT101] Phone temperature: “Phone gets really hot and they find it difficult to get a reading- kind of frustrating using the HRV” [HRT104]
	Usability: 8	Perceptions of the capacity of the system to perform tasks safely, effectively, or efficiently while enjoying the experience	“I think it’s complicated with having to choose between baseline and feedback, I would like it to be simplified.” [HRT110]

### Coaching Interview Findings

Participant feedback on both the initiation and feedback coaching components were similar. The participants overwhelmingly liked the coaching style n=11 and reported benefiting from the coach using different behavior change strategies (ie, n=17: goal setting, social support, and providing information; Table 8). One participant felt that the supportive coach helped with their ability to cope with stress, whereas another felt that the added accountability helped keep them on track with their goals. There were no negative responses regarding the BCTs used by the counselors. However, 1 participant did not like the motivational interviewing–based counseling style, suggesting that they wanted the coach to be more directive in providing exercise during the session. A total of 4 respondents suggested that 15-minute sessions were not long enough for coaching to facilitate health behavior changes. Coaching was conducted web-based through the CoreHealth platform, and there were 4 mentions of difficulty in knowing where and how to access videoconferencing.

**Table 8:** Participant interview findings related to coaching conditions.

Interview findings and code		Definition	Illustrative quote
**Likes, 5 codes**			
	BCT^a^: 17 Subcodes: Goal setting: 5 Social support: 11 Health-related information: 1	Goal setting: setting or agreeing on a goal defined in terms of the behavior to be achieved Unspecified social support: providing resources (eg, psychological) intended to benefit an individual’s ability to cope with stress [64] Health-related information: providing health-related information to the participant	Goal setting: “Setting up, setting up the objective, making sure you’re on the right track, accountability.” [HRT108] Social support: “She applied all the great practices right, the not, no judgment, supporting... Uh, so look, you know a potential solutions and make you know, just raising ideas, bringing motivation” [HRT108] Health-related information: “She understood activity and exercise and all of these things that we talked about it. I mean it was just being familiar with the content on her part was really good.” [HRT106]
	Coaching style: 13	Perceived like or dislike of the coaching style	“The thing I liked was it was really kind of nice to talk to a live friendly voice. She was very friendly, very positive, very upbeat.” [HRT111]
**Dislikes, 3 codes**			
	Counseling length: 4	Perceived like or dislike of the counseling session duration	“[I] dislike the length of it. If you’re going to put the effort in, probably to have someone help you, I think 15 minutes is personally too short.” [HRT115]
	Technical problems: 4	Perceived challenges associated with using the CoreHealth platform for the web-based coaching session	“When I was trying to log in the second time, I’m like how did I do it the first time and I just like totally forgot.... Uh, so we got my gosh I’m going to be late.” [HRT107]
	Coaching style: 2	Perceived like or dislike of the coaching style	“So it was just her agreeing with me which I don’t know if you like or do not like I think I was expecting it to act like her don’t like a strategy that I had heard over like something that I didn’t think of...” [HRT115]

^a^BCT: behavior change technique.

### Interview With the Wellness Coach

Overall, the coach was satisfied with the coaching and found that using the spirit of motivational interviewing was beneficial to bridge the gap and allowed the participants to connect to them as a coach. The coach stated that “Motivational interviewing is definitely one that I use a lot.” Although 15 minutes is the standard length of counseling for most low-risk clients, they mentioned that “15 minutes is a tight time frame to be able to really utilize a lot of motivational interviewing tools*.”* They also suggested that stress and health behavior change content should be separated and introduced at different time points. The coach mentioned that the study’s setup and participants receiving different conditions was a bit confusing. They mentioned the following:

The communication was probably the hardest part is being able to get in touch with the participants and make sure that they’re on track...I’m used to being that person that goes and holds their hand and helps them along the entire way.

This seemed particularly the case for those receiving feedback counseling but not initiation counseling, where the first communication between the coach and participant occurred part-way through the program. The coach also echoed the challenges that were heard from participants in connecting Light Heart to CoreHealth to view their HRV-BF in CoreHealth as follows:

I think that’s the main thing is making sure people are utilizing it [Light Heart] properly. And then because, I mean, when people did use it, I received the information in CoreHealth. So, if the information is there, it’s really easy to be able to coach on. It’s just making sure that they see their end.

## Discussion

### Principal Findings

We conducted this study to assess participants’ perceptions of different levels of the intervention components to be tested in a future optimization stage. There were many positive perceptions about the intervention components; however, interviews with participants and the coach revealed areas of strength and improvement.

In general, participants expressed more positive than negative reactions toward the onboarding process, consistent with the quantitative acceptability scores. Although some participants found it easy to learn the CoreHealth site and study apps, others suggested that additional resources were needed to help individuals engage with the technology as they started the program (eg, instructional videos). There were also mixed responses regarding the length of the 8-week program. Although some felt that it was right, others found it too long. Previous literature has suggested that sequentially initiating changing to multiple behaviors (ie, 4 weeks of health behavior change followed by 4 weeks of stress management) may be more effective than having participants concurrently initiate changes in multiple behaviors at the same time [65]. This, combined with the feedback from participants and the coach to separate the stress management content from the health behavior change content, will result in a disaggregation of the content into 2 separate 4-week modules. Notably, there were no differences between delivering feedback coaching at either 2 or 4 weeks. Moving forward, coaching will be delivered at the 4-week time point to align with the updated structure of delivering two 4-week modules.

In reporting what they found helpful about the 8-week program, participants frequently mentioned 6 different BCTs. These responses are an indication of treatment receipt fidelity—that participants received the BCTs on the CoreHealth platform as intended [66]. One exception was the action planning content, which participants found cumbersome to use. The quality of how interventions were implemented using technology could impact the amount of time it took to complete a task [67]. Asking participants to set weekly action plans that were time-consuming may have negatively affected their experience. We plan to work with developers to simplify the action planning script to improve the user experience because health information technology systems that are easy to navigate are more likely to be used [68,69]. We also plan to work with developers to improve the process of connecting the Light Heart and CoreHealth apps to the CoreHealth Web platform through their mobile access codes.

Participants enjoyed the look and feel of the Light Heart app and reported important benefits in obtaining HRV. There were 3 noted areas for improvement related to our integration of the app within this study. First, incorporating the HRV function from Light Heart into the broader CoreHealth app would enhance usability and ease the burden of having to switch between using 2 different apps. Combining the 2 apps would also alleviate the second challenge, which is the difficulty of connecting the apps through mobile phone codes. Third, providing additional instructional resources may help alleviate other challenges that participants experienced when first learning to use the app (eg, finger placement). We believe that making these adjustments will improve the usability and acceptability of the study’s apps.

Despite these challenges, participants reported the benefits of using Light Heart to obtain HRV-BF. Biofeedback has been shown to reduce stress and improve well-being [70]. Consistent with these findings, 1 participant in the momentary HRV-BF group explicitly noted that the biofeedback improved their mindfulness and physical feeling. Biofeedback is a form of self-monitoring. Regular self-monitoring can prime individuals to think about their behavior change goals, particularly for those who do not regularly self-monitor [71]. One participant in the daily resting HRV-BF group suggested that taking regular readings served as a regular reminder of their behavior change goals. These self-reported benefits from the 2 HRV-BF conditions provide an anecdotal indication that the participants received their HRV-BF condition and obtained the hypothesized effects as intended.

One of the key activities of the preparation phase is to pilot the candidate components and gather evidence to build a conceptual model before the optimization phase [50,51]. The initial intervention components and levels were determined in consultation with CoreHealth staff based on their affordability and scalability because they align with their current standard practices. Participants’ feedback on the candidate components provided insight into potential mediators to include and assess in a future conceptual model. For example, feedback about HRV-BF did not substantially differ between the daily resting and momentary components, and the findings pointed to increased awareness of current HRV as a potential mediator. The participants suggested that using different BCTs during coaching helped them track and achieve their wellness goals. This may suggest self-regulatory efficacy in managing one’s health behaviors as a potential mediator of coaching components. We plan to review the candidate components and mediators to develop a conceptual model before the next phase of this project.

### Strengths

A notable strengths of the study was using a multimethod approach used in this preparation phase and adhering to the MOST framework. For example, obtaining user feedback answers calls to include participants in developing and refining health technology [68]. MOST is a progressive framework for the development and optimization of behavioral treatments. Framing this study using MOST provided clear benchmarks for this study’s objectives and a clear path to progress to the next stage of research. Research can be more impactful and have better patient outcomes when key stakeholders are meaningfully engaged [72,73]. One final strength was the partnered approach used to execute this study. Throughout this project, we had a close working relationship with the CoreHealth staff, which allowed for the quick resolution of technological challenges as they arose. For example, we were able to find a quick solution to the challenge of mobile phone codes not working properly at the beginning of the study. Partnered research also increases the likelihood that the findings will be put into practice [74]. The findings from this study will result in changes that are implemented by our research partner. Two such examples include adjusting the action plan widget and adjusting how users navigate between the 2 types of HRV-BF readings in the Light Heart app.

### Limitations

One possible limitation of conducting research with multiple partners was the continued reliance on all partners to perform their roles to ensure the ongoing success of a project. We had exceptional buy-ins from both CoreHealth and our corporate coaching partner throughout this study. However, owing to the economic impact of the COVID-19 pandemic, our coaching partner is no longer able to deliver health coaching. This possibility was anticipated at the onset of our research with CoreHealth, which indicated that they work with other health coaching companies that can be approached to run the next phase of the study. Another limitation was that the interview questions may not have been sensitive enough to distinguish between similar HRV-BFs or coaching components. One aim of preparation studies is to gain insight into the delivery of candidate components and different conditions. Future preparation phase research should seek more nuanced feedback about the receptivity of the different component combinations in the individual conditions. Another limitation of this study was the dropout rate, which was higher than anticipated, yielding fewer participant responses to our poststudy assessments. These adherence rates are consistent with other lifestyle intervention trials reporting up to 70% attrition, depending on the length of the follow-up [75-77]. Multiple participants received each of the 4 candidate components; however, 4 (25%) of the 16 conditions did not receive participant feedback and went unpiloted because of dropout.

MOST is a structured framework for developing efficient behavior change interventions. This preparation study will result in improved onboarding and app usability. Given that there were unpiloted conditions, proposed changes based on user feedback, and changes in corporate wellness providers, we plan to reiterate through the preparation phase before proceeding to the optimization phase. Strong partnerships with a commitment to make user-centered adjustments will allow us to keep progressing through the different phases of MOST.

## Abbreviations

BCT: behavior change technique
HRV: heart rate variability
HRV-BF: heart rate variability biofeedback
MOST: multiphase optimization strategy
sdNN: SD of the normal sinus beats

## Data Availability

Deidentified data may be made available by reasonable request to the corresponding author.
